# Hypoxia Activates a Ca^2+^-Permeable Cation Conductance Sensitive to Carbon Monoxide and to GsMTx-4 in Human and Mouse Sickle Erythrocytes

**DOI:** 10.1371/journal.pone.0008732

**Published:** 2010-01-15

**Authors:** David H. Vandorpe, Chang Xu, Boris E. Shmukler, Leo E. Otterbein, Marie Trudel, Frederick Sachs, Philip A. Gottlieb, Carlo Brugnara, Seth L. Alper

**Affiliations:** 1 Molecular and Vascular Medicine Unit, Beth Israel Deaconess Medical Center, Harvard Medical School, Boston, Massachusetts, United States of America; 2 Renal Division, Beth Israel Deaconess Medical Center, Harvard Medical School, Boston, Massachusetts, United States of America; 3 Department of Surgery, Beth Israel Deaconess Medical Center, Harvard Medical School, Boston, Massachusetts, United States of America; 4 Department of Laboratory Medicine, Children's Hospital Boston Harvard Medical School, Boston, Massachusetts, United States of America; 5 Department of Medicine, Harvard Medical School, Boston, Massachusetts, United States of America; 6 Department of Surgery, Harvard Medical School, Boston, Massachusetts, United States of America; 7 Department of Pathology, Harvard Medical School, Boston, Massachusetts, United States of America; 8 Institut de Recherches Cliniques de Montreal, Montreal, Quebec, Canada; 9 Department of Physiology and Biophysics, University of Buffalo, Buffalo, New York, United States of America; Duke University, United States of America

## Abstract

**Background:**

Deoxygenation of sickle erythrocytes activates a cation permeability of unknown molecular identity (Psickle), leading to elevated intracellular [Ca^2+^] ([Ca^2+^]_i_) and subsequent activation of K_Ca_ 3.1. The resulting erythrocyte volume decrease elevates intracellular hemoglobin S (HbSS) concentration, accelerates deoxygenation-induced HbSS polymerization, and increases the likelihood of cell sickling. Deoxygenation-induced currents sharing some properties of Psickle have been recorded from sickle erythrocytes in whole cell configuration.

**Methodology/Principal Findings:**

We now show by cell-attached and nystatin-permeabilized patch clamp recording from sickle erythrocytes of mouse and human that deoxygenation reversibly activates a Ca^2+^- and cation-permeable conductance sensitive to inhibition by *Grammastola spatulata* mechanotoxin-4 (GsMTx-4; 1 µM), dipyridamole (100 µM), DIDS (100 µM), and carbon monoxide (25 ppm pretreatment). Deoxygenation also elevates sickle erythrocyte [Ca^2+^]_i_, in a manner similarly inhibited by GsMTx-4 and by carbon monoxide. Normal human and mouse erythrocytes do not exhibit these responses to deoxygenation. Deoxygenation-induced elevation of [Ca^2+^]_i_ in mouse sickle erythrocytes did not require KCa3.1 activity.

**Conclusions/Significance:**

The electrophysiological and fluorimetric data provide compelling evidence in sickle erythrocytes of mouse and human for a deoxygenation-induced, reversible, Ca^2+^-permeable cation conductance blocked by inhibition of HbSS polymerization and by an inhibitor of strctch-activated cation channels. This cation permeability pathway is likely an important source of intracellular Ca^2+^ for pathologic activation of KCa3.1 in sickle erythrocytes. Blockade of this pathway represents a novel therapeutic approach for treatment of sickle disease.

## Introduction

Sickle cell disease is caused by the homozygous missense mutation of Glu to Val in codon 6 of the hemoglobin (Hb) β chain gene, encoding the mutant βS globin polypeptide. In the absence of wildtype β globin, assembly of tetrameric α_2_βS_2_ generates sickle hemoglobin (HbSS). Polymerization of deoxy-HbSS leads to oxidation, crosslinking, stiffening, and distortion of the red cell membrane, increased adhesiveness to leukocytes and to endothelial cells, and cell lysis. The resulting hemolytic anemia and diffuse vaso-occlusive pathology causes life-long illness for which the only currently approved chronic drug treatment is hydroxyurea [1], supplementing the traditional mainstays of symptomatic treatment: hydration, pain relief, anti-inflammatory drugs, and antibiotics. Marrow transplantation can be curative, but the associated morbidity remains high enough to discourage its widespread use even in developed countries. Although gene therapy continues to show promise, development of adjunct pharmacotherapy remains a high priority for treatment and management of sickle disease [2].

Mature human sickle red cells (SS cells) are dehydrated by inappropriate hypoxic activation of erythroid K-Cl cotransporters and of the erythroid Ca^2+^-activated K^+^ channel KCa3.1/IK1/KCNN4, also known as the “Gardos channel” [3], [4], [5], [6]. The consequent elevation of intracellular [HbSS] dramatically shortens the “delay time” [7] for assembly of the critical aggregate of deoxy-HbSS tetramers required for subsequent rapid growth of deoxy-HbS fiber length [8]. Therapeutic prolongation of the deoxy-HbSS delay time is the goal of pharmacological inhibition of sickle erythrocyte solute leak and dehydration [4], [9]. A study of inhibition of erythroid K-Cl cotransporters with magnesium pidolate is emerging from Phase I [10]. The KCa3.1 inhibitor ICA-17043 (senicapoc) completed Phase II clinical trial with promising results [11] and progressed through much of Phase III with continued, convincing hematological efficacy, although without improvement in clinical pain symptoms [12]. Endothelin antagonists recently shown to be of benefit in mouse models of sickle disease [13] likely exert their effect through KCa3.1 inhibition and consequent reduction of erythrocyte dehydration[14].

The elevated cytosolic [Ca^2+^] ([Ca^2+^]_i_) required to activate KCa3.1 is elicited by deoxygenation in SS cells but not in normal (AA) cells. Inhibition of this nonspecific cation permeability of yet unknown molecular identity [9], [15], referred to as Psickle, should in theory synergize with senicapoc in the treatment of sickle disease. Whole cell currents recorded in symmetrical Na^+^ solutions from SS cells were increased by deoxygenation and displayed properties suggestive of Psickle, including partial inhibition by 4,4′-diisothiocyanatostilbene-2,2′-disulfonic acid (DIDS), Zn^2+^, and Gd^3+^ [16]. However, in the asymmetric conditions of pipette NMDG and bath NaCl, deoxygenation did not significantly increase whole cell inward current or decrease outward current in SS cells. Deoxygenation of SS cells in symmetrical Ca^2+^ solutions also produced no significant increase in whole cell current [16]. Thus, one of the central properties of Psickle, a Ca^2+^-permeable cation current activated by deoxygenation, remained undetected in the sickle red cell membrane.

We now report hypoxic activation of currents recorded from cell-attached patches of intact SS cells. Deoxygenation-induced currents displayed properties consistent with those of Ca^2+^-permeable cation channels. Activation of these currents required sickle hemoglobin polymerization, as evidenced by their inhibition by prior carbon monoxide exposure. Deoxygenation elevated [Ca^2+^]_i_ in parallel with conductance activation, and with similar pharmacological properties.

Psickle has not previously been described in red cells from mouse models of sickle disease. We found red cells from two mouse models of sickle disease to exhibit deoxygenation-induced currents and [Ca^2+^]_i_ elevation with inhibitor sensitivity similar to that in human SS cells. Deoxygenation-induced currents in SAD mouse red cells were completely reversible. SAD mouse red cell membrane patches sustained GΩ seals during transitions from room air to nitrogen, and then back to room air. The initial elevation of [Ca^2+^]_i_ elicited by deoxygenation did not require KCa3.1 channel activity, but later phases of [Ca^2+^]_i_ elevation were attenuated by absence of KCa3.1 and enhanced by inhibition of the plasmalemmal Ca^2+^ ATPase (PMCA). Human AA red cells and normal mouse red cells exhibited no deoxygenation-induced increases in current or in [Ca^2+^]_i_.

These observations extend the phenotypic characterization of deoxygenation-activated Ca^2+^ transport in human sickle red cells, and present the first such data in mouse sickle red cells. The observations include realtime, on-cell and whole cell patch clamp evidence of deoxygenation-induced elevated Ca^2+^ and cation conductance, flourimetric evidence of elevated [Ca^2+^]_I_, and description of novel inhibitors of these Psickle-like activities.

## Results

### Deoxygenation Reversibly Increases Conductance in Membranes of SAD Cells

The SAD mouse expresses a relatively mild form of sickle cell anemia [17] with many of the longterm pathophysiological complications of chronic sickle disease in humans [18]. The SAD transgene encodes human α-globin and a triply mutant, hypersickling human Hb β chain, generating ∼20% Hb SAD with continued expression of mouse Hbb chains [17]. Our current colony, now at 18 years' remove from the founders, exhibits minimal anemia or reticulocytosis in the adult mouse [5], but retains a robust phenotype of cell dehydration. Psickle has not previously been characterized in mouse models of sickle disease.

**Figure 1:**
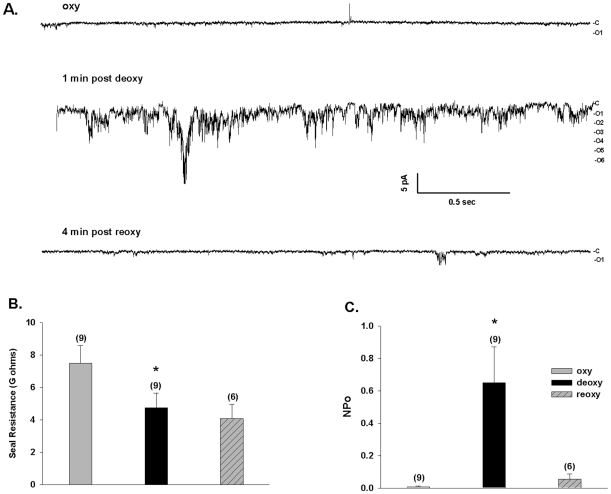
Deoxygenation reversibly activates a conductance in red cells from SAD sickle mice. A. Representative current trace from an individual cell-attached patch on a SAD sickle mouse erythrocyte before deoxygenation (upper trace, oxy), 1 min post-deoxygenation (middle trace, deoxy), and 4 min post-reoxygenation (lower trace, reoxy); −Vp  = −50 mV. Symmetric pipette and bath solutions contained (in mM) 140 NaCl, 4 KCl, 1CaCl_2_, 1 MgCl_2_, 10 Na HEPES, pH 7.4. B. Seal resistance was maintained during deoxygenation in all 9 cells, and during reoxygenation in 6 of the 9 cells (*, p<0.01 vs. oxy; p =  N.S. vs. re-oxy, ANOVA). C. Summary of patches such as in panel A, showing that the increased NPo (product of the number of single channels and the channel open probability) observed after deoxygenation was reversible (without change in seal resistance) upon reoxygenation (*, p<0.02 vs. oxy; p<0.05 vs. reoxy, ANOVA). Values are means ± s.e.m.

The on-cell patch records of Figure 1A, with NaCl in both pipette and bath, show that deoxygenation activates noisy channel activity in the SAD sickle mouse red cell membrane. Deoxygenation increased SAD red cell patch NPo from the room air value of 0.01±0.01 to 0.65±0.22 (n = 9). Subsequent reoxygenation decreased nPo to 0.06±0.03 in those patches that survived (n = 6; Figure 1C). Calculated chord conductance was 20 pS (between −Vp = 0 and −50 mV; n = 9). Visual inspection of current traces indicated onset of increased activity at 7.5±3.1 sec after deoxygenation (n = 9; measured from the bath solution change artifact). The nearly complete cessation of channel activity upon re-oxygenation was accompanied by maintenance of a stable giga-ohm seal (Figure 1B). The time to steady-state recovery of quiescence after re-oxygenation was 57±20 sec (n = 6). Thus, the noisy nature of the deoxygenation-induced conductance in on-cell patches did not reflect deoxygenation-induced leak due to loss of the original tight seal.

### Deoxygenation of SAD Sickle Mouse Red Cells Increases Cation Conductance and Elevates [Ca^2+^]_i_

**Figure 2:**
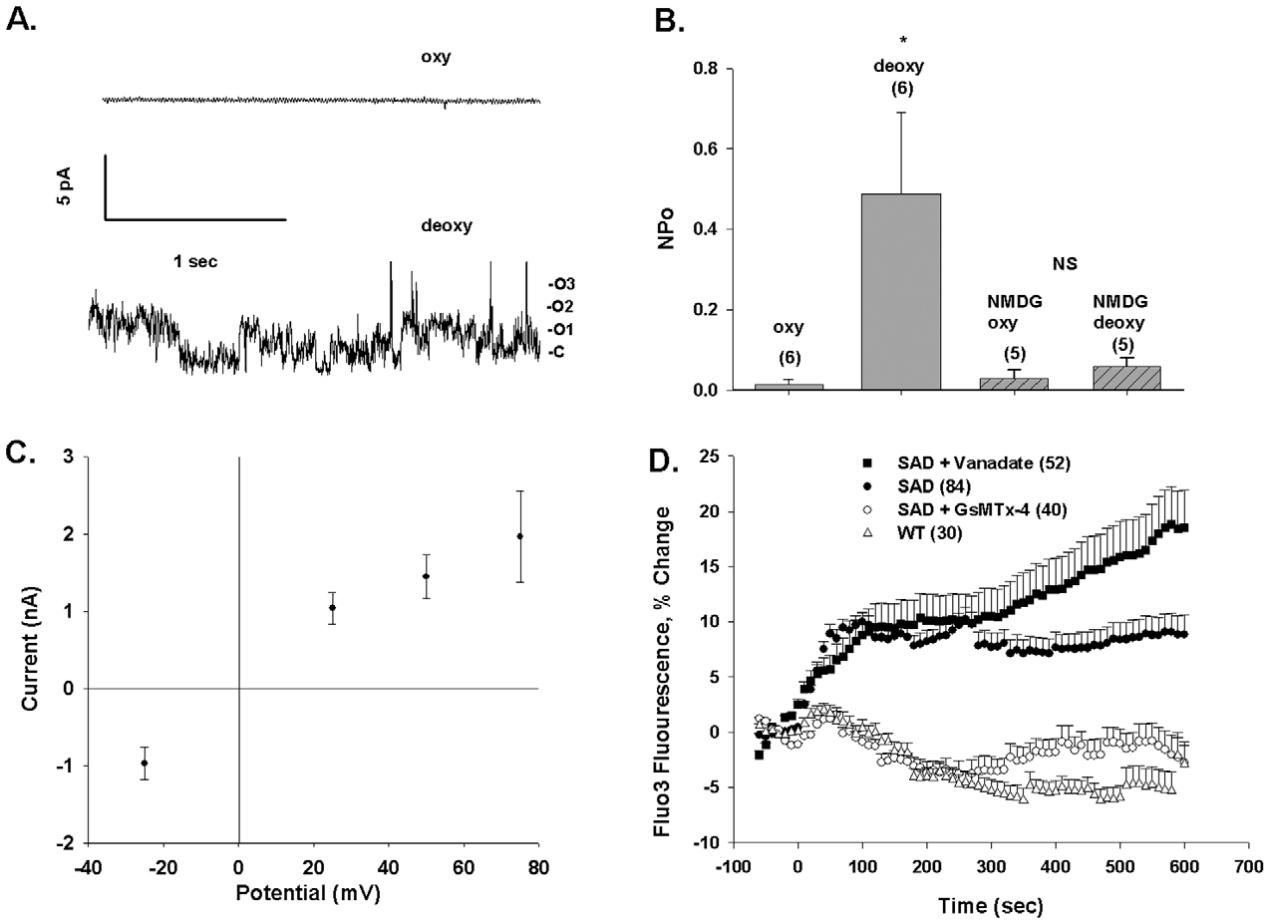
Deoxygenation activates conductance and increases [Ca^2+^]_i_ in red cells from SAD sickle mice. A. Representative current trace from an individual cell-attached patch on a SAD mouse erythrocyte, recorded first in oxygenated (upper trace, oxy, −Vp = −25 mV) and subsequently in deoxygenated conditions (lower trace, deoxy, −Vp = +75 mV). Symmetrical pipette and bath solutions contained (in mM) 150 Na methanesulfonate, 10 Na EDTA, and 10 Na HEPES, pH 7.4. B. Deoxygenation increased the NPo of 6 cell-attached patches recorded in symmetrical Na methanesulfate (*, p<0.05; n = 6). Substitution of pipette solution Na^+^ with NMDG blocked the deoxygenation-induced increase in NPo (n = 5). Values are means ± s.e.m, recorded at −Vp = −25 mV. C. Current-voltage relationship in a representative cell-attached patch on a SAD red cell exposed to deoxygenation with symmetrical Na methanesulfonate solutions in pipette and bath. Mean ± s.e.m. for fit of the amplitude histogram. D. Deoxygenation increases [Ca^2+^]_i_ in SAD red cells but not in WT mouse red cells, in a manner inhibited by 1 µM GsMTx-4 and enhanced by 50 µM vanadate. Values are means ± s.e.m. of Fluo-3 fluorescence increase for (n) red cells from 3 mice studied in 8 experiments (SAD), from 1 mouse studied in 4 experiiments (WT and SAD + GsMTx-4) or from 1 mouse studied in 2 experiments (SAD + vanadate).

To minimize the contribution of anion currents to measured currents, and to enhance detection of monovalent cation currents, the effects of deoxygenation in SAD cells were studied in a Na methanesulfonate bath free of Ca^2+^. The pipette also contained Na methanesulfonate. The representative on-cell patch shown in Figure 2A was quiescent in room air, but upon deoxygenation exhibited channel activity with a chord conductance of 27 pS (between −Vp = +25 and −25 mV) and reversal potential (E_rev_) of +1 mV (Figure 2C). As shown in Figure 2B, deoxygenation increased mean NPo in SAD mouse red cell patches from 0.01±0.013 to 0.48±0.20 (n = 6; p<0.05). Patch duration under deoxygenation in these experiments was 8.4±1.8 min. Estimated single channel amplitude at −Vp = 25 mV was 0.70±0.14 pA (n = 6), with a calculated inward chord conductance of 28 pS (0 to −25 pS). With NMDG chloride rather than Na methanesulfonate in the pipette, room air patch NPo of 0.03 was unchanged by deoxygenation at 0.06 (n = 5, P = 0.28). The data demonstrate the activation by deoxygenation of nonspecific cation channel activity in the SAD mouse red cell membrane.

**Figure 3:**
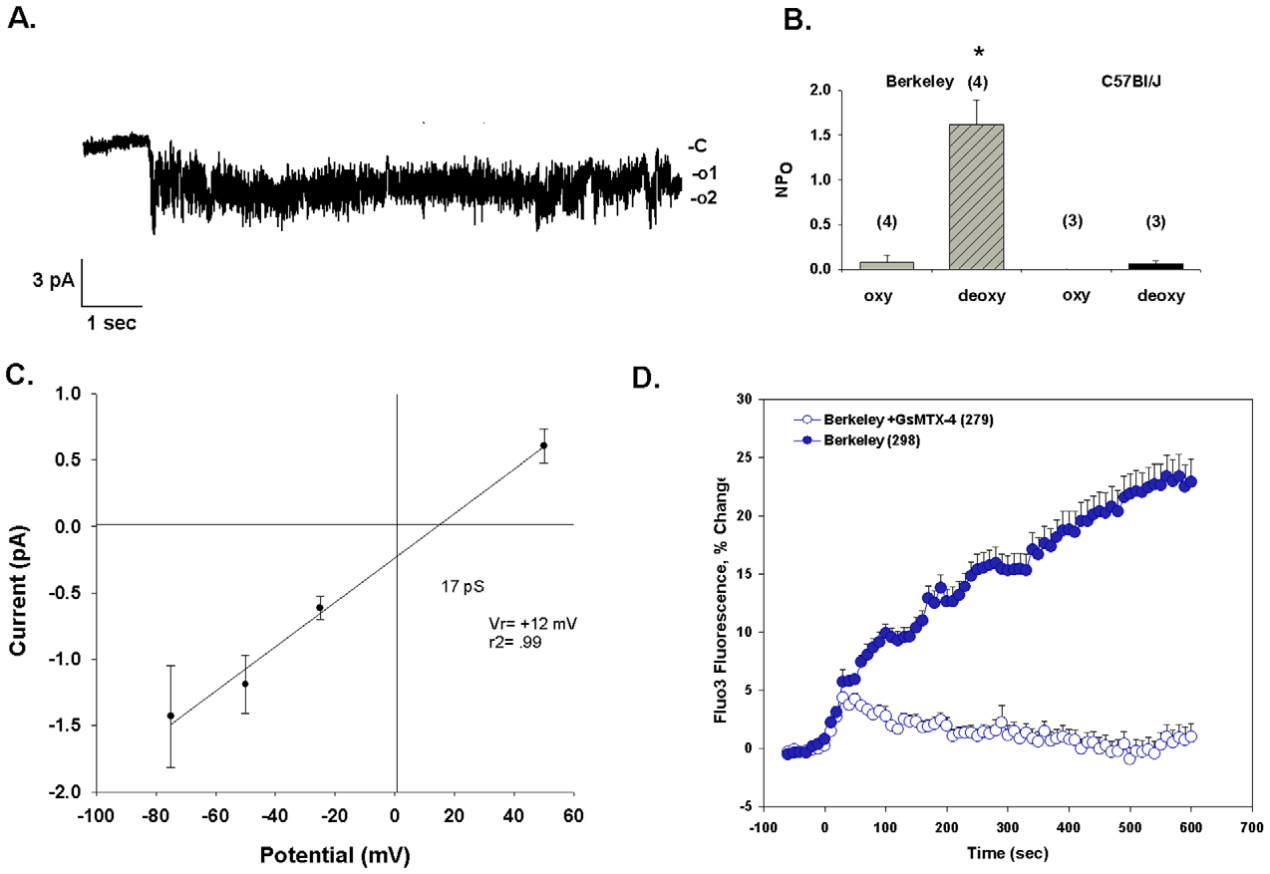
Deoxygenation activates conductance and increases [Ca^2+^]_i_ in red cells from Berkeley sickle mice. A. Representative current trace from an individual cell-attached patch on a Berkeley sickle mouse erythrocyte. Symmetrical pipette and bath solutions contained (in mM) 150 Na methanesulfonate, 10 Na EDTA, and 10 Na HEPES, pH 7.4. The cell was subjected to deoxygenation at t = 0. −Vp = −25 mV. B. Deoxygenation increased in Berkeley mouse cells (n = 4), but not in C57BL6/J cells (n = 3). Values are means ± s.e.m, recorded at −Vp = −25 mV. C. Current-voltage relationship of channel activity actived by deoxygenation in Berkeley red cells. Mean ± s.e.m. for fit of the amplitude histogram. D. Deoxygenation-increased [Ca^2+^]_i_ in Berkeley red cells (9 experiments) was prevented by preincubation with 1 µM GsMtx-4 bath preincubation (7 experiments). Values are means ± s.e.m. of Fluo-3 fluorescence increase for (n) red cells from 3 mice.

The complete bath chloride replacement conditions used in the experiments of Figure 2 (as well as in Figures 3 and 6) were shown previously in human AA red cells to alkalinize pH_i_ to steady-state values of 7.7 with gluconate substitution [19], [20], to 7.5 with phosphate substitution [19], and to 7.8–8.1 with citrate substitution [21], [22]. Although such intracellular alkalinization might modify erythroid membrane channel activities, deoxygenation comparably activated conductance in SAD cell membrane patches whether in the presence of methanesulfonate or chloride in the pipette and bath (Figs. 1 and 2).

Since the proximate pathological consequence of Psickle activation is believed to be elevation of SS cell [Ca^2+^]_i_, we monitored Fluo-3 fluorescence emission during deoxygenation in SAD cells. Deoxygenation was found to increase [Ca^2+^]_i_ in SAD red cells (P<10^−5^) but not in red cells from the parental strain C57Bl6/J (Figure 2D). The increased [Ca^2+^]_i_ accompanying deoxygenation was completely inhibited by the selective blocker of stretch-activated ion channels, GsMTx-4 (1 µM, P<10^−5^) [23], [24]; [but see also [25], [26], [27]]. [Ca^2+^]_i_ elevation over the first 5–10 min after deoxygenation occurred without significant decrease in mean corpuscular volume (MCV; L. de Franceschi, personal communication). This suggests that a major proportion of the increased non-ratiometric fluorescence emission of Fluo-3 did not merely reflect increased intracellular dye concentration (secondary, in any case, to cell shrinkage triggered by entry of extracellular Ca^2+^).

The role of red cell plasma membrane Ca^2+^-ATPase (PMCA) in controlling the maximal value of [Ca^2+^]_i_ induced by deoxygenation was tested by bath addition of 50 µM Na vanadate (Figure 2D), a concentration sufficient for near-complete inhibition of human red cell PMCA [28], [29]. During 5 min normoxic vanadate preincubation of SAD cells, [Ca^2+^]_i_-dependent Fluo-3 emission increased 12.8±1.8%. Acute deoxygenation in the continued presence of vanadate further elevated SAD red cell [Ca^2+^]_i_, with kinetics similar to those in the absence of vanadate, achieving plateau values within <2 min. However, after ∼4 min longer at this plateau value, vanadate-exposed cell [Ca^2+^]_i_ slowly increased to twice the previous plateau value (Figure 2D). This late elevation might reflect PMCA inhibition by ATP depletion (the bath solution lacks pyruvate or glucose), or recruitment of the distinct, vanadate-induced Ca^2+^ entry pathway previously identified in human AA cells [30].

### Deoxygenation of Berkeley Sickle Mouse Red Cells Increases Cation Conductance and Elevates [Ca^2+^]_i_

The Berkeley sickle mouse exhibits severe hemolytic anemia resembling humans with sickle-β-thalassemic disease [31]. Figure 3A shows a representative current trace from a cell-attached patch on a Berkeley mouse erythrocyte subjected to deoxygenation. Total seal duration in this individual experiment was 6.5 min, including 2 min in room air. Conductance was abruptly activated by deoxygenation after 1.1 sec in this experiment. However, the mean deoxygenation time prior to activation of conductance was 18±11 sec (n = 5). Deoxygenation increased NPo from 0.081±0.080 to 1.62±0.28 in Berkeley sickle red cells (n = 4, p<0.01), whereas NPo of oxygenated C57Bl6/J red cells (0.001±0.001) was only minimally increased by deoxygenation (0.057±0.038; n = 3, Figure 3B). Estimated single channel amplitude of deoxygenation-induced channels with Na methanesulfonate in pipette and bath was 0.62±0.06 pA at −Vp = −25 mV. Slope conductance in this patch (between −Vp = −75 and +50 mV) was 17 pS conductance with a reversal potential of +12 mV (Figure 3C). Mean initial seal resistance of 1.8±0.2 GΩ in room air (n = 5) was maintained under deoxygenated conditions, and just before loss of seal was 2.3±0.4 GΩ. Deoxygenation also increased Berkeley red cell [Ca^2+^]_i_ (P<10^−5^), and this [Ca^2+^]_i_ elevation was inhibited nearly completely by 1 µM GsMTx-4 (P<10^−5^; Figure 3D).

### Deoxygenation Increases [Ca^2+^]_i_ in SS Cells

**Figure 4:**
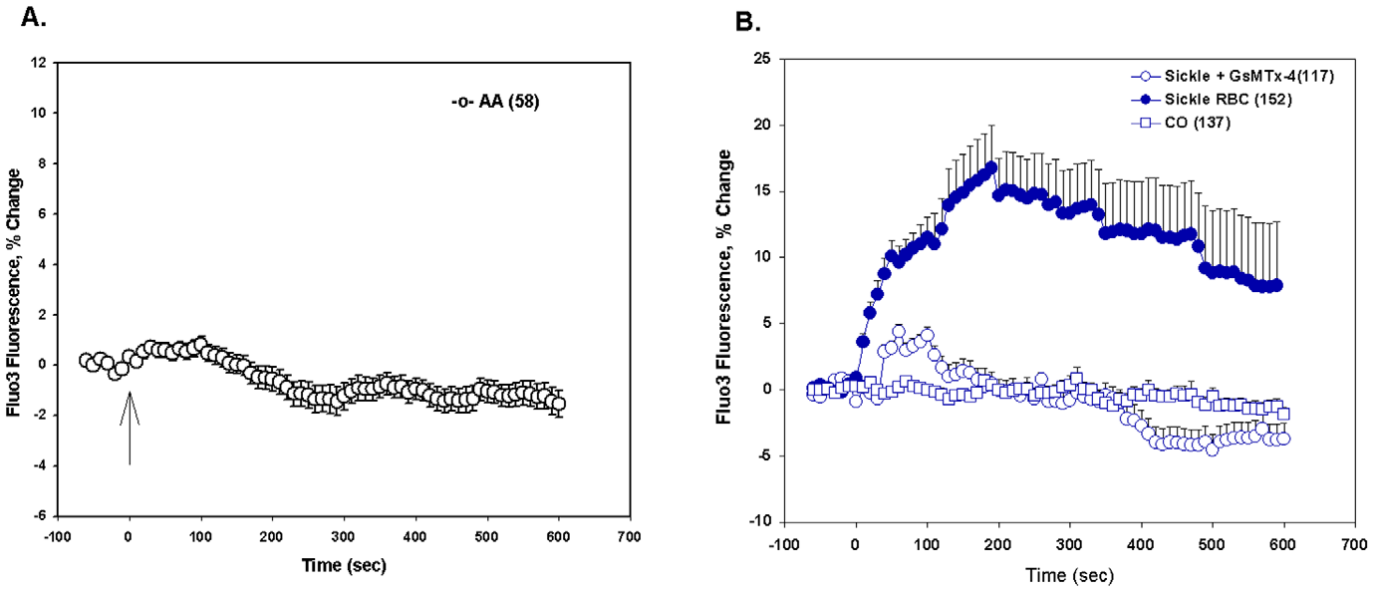
Deoxygenation elevates [Ca^2+^]_i_ in human SS red cells but not in human AA red cells. A. Fluo-3-loaded AA cells exposed to deoxygenation at t = 0 (arrow) did not exhibit increased fluorescence, indicating lack of increase in [Ca^2+^]_i_. Values are means ± s.e.m. from AA cells (n = 58) from 2 subjects, as acquired during six experiments. B. Fluo-3-loaded human SS cells responded to deoxygenation with elevation of [Ca^2+^]_i_ to peak, sustained values within 2–3 min (filled circles, SS cells (n = 152) from 4 subjects, examined in 8 experiments). This increase was blocked by inclusion of 1 µM GsTMx-4 in the bath (open circles, SS cells (n = 117) from 3 subjects, examined in 4 experiments) or by prior treatment with CO as described in Methods (open squares, SS cells (n = 137) from 3 subjects, examined in 5 experiments). See Figure S1 for fluorescence intensities of individual cells of each genotype at single time points of maximal [Ca^2+^]_i_ elevation.

Deoxygenation of human sickle red cells has previously been noted to increase ^45^Ca^2+^ influx [9], [15], [32], [33], [34], [35]. As shown in Figure 4, deoxygenation also increased [Ca^2+^]_i_ in Fluo-3-loaded human SS red cells [(P<0.001, Mann-Whitney test or unpaired two-tailed T test (Figure 4B) but not in AA cells (Figure 4A)]. The distribution of individual cell [Ca^2+^]_i_ values shows that not all SS cells responded to deoxygenation by elevating [Ca^2+^]_i_ (Figure S1). 10 min pretreatment of SS cells with 1 µM GsMTx-4 inhibited [Ca^2+^]_i_ elevation by subsequent deoxygenation in continued presence of the peptide (P<0.001, Figure 4B). 30 min preincubation with CO also prevented [Ca^2+^]_i_ elevation by deoxygenation (in the absence of continued CO exposure) (P<0.001, Figure 4B). These results suggested that deoxygenation-activated elevation of [Ca^2+^]_I_ in SS cells required both HbS polymerization and activation of a (pharmacologically defined) stretch-activated ion conductance. The magnitude of the deoxygenation-induced increase in SS cell [Ca^2+^]_i_ was considerably lower than that elicited in SS cells by 5 µM LPA (Figure S2C), previously shown to increase [Ca^2+^]_i_ in AA red cells [36].

### Deoxygenation Activates a Ca^2+^-Permeable Conductance in Cell-Attached Patches of Human SS Cells

**Figure 5:**
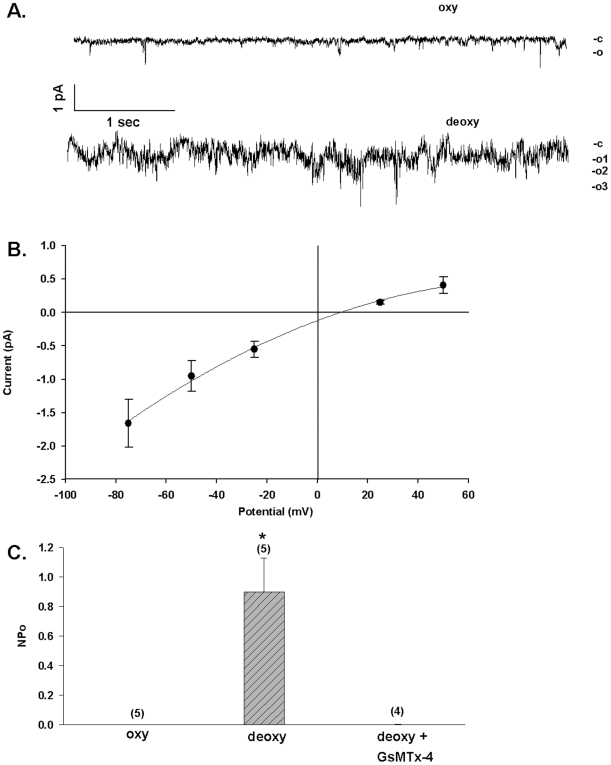
Deoxygenation activates a Ca^2+^-permeable conductance in human SS cells. A. Representative current traces from a cell-attached patch on an individual human SS erythrocyte recorded before (oxy) and after onset of deoxygenation (deoxy). Pipette solution contained (in mM) 100 CaCl_2_, 10 Na HEPES, pH 7.4. Bath solution contained (in mM) 150 Na methanesulfonate, 10 Na HEPES, pH 7.4. Holding potential was −Vp = −25 mV. Open states at right are derived from the open state histogram (not shown). B. Current-voltage relationship derived from the deoxygenated currents measured in the patch of panel A. Mean ± s.e.m. for fit of the amplitude histogram. C. The low NPo of inward single channel activity of human SS cells recorded in the on-cell configuration with Ca^2+^ in the pipet is increased by deoxygenation (*, P<0.02). The deoxygenation-induced increase in NPo is prevented by inclusion of 1 µM GsMTx-4 in the pipette. Values are means ± s.e.m. (n = 4–5), recorded at −Vp = −25 mV.

Ca^2+^ entry into deoxygenated SS cells via Psickle is believed to be a major trigger of SS cell dehydration via KCa3.1. However, in a previous report [16] deoxygenation did not increase whole cell currents recorded in human SS cells in symmetrical Ca^2+^ solutions. We therefore sought electrophysiological evidence of deoxygenation-activated Ca^2+^ permeation in cell-attached patches with CaCl_2_ in the pipette and Na methanesulfonate in the bath. As shown in Figures 5A and 5C, deoxygenation increased patch conductance. Patch NPo was not different from zero in room air, but increased to 0.90±0.23 upon deoxygenation (P<0.05; n = 5). Mean deoxygenation time prior to activation of conductance was 21±10 sec. The induced current displayed moderate inward rectification in these conditions, with amplitude of 0.64±0.089 pA at −Vp =  −25 mV (n = 5), corresponding to a 25 pS chord conductance. The I-V curve of the Figure 5A patch, with E_rev_ of +7 mV (Figure 5B) was consistent with an inward Ca^2+^ current and a substantial fraction of outward K^+^ current through a nonspecific cation conductance. The calculated E_rev_(Cl^−^), −18 mV for ∼100 mM [Cl^−^]_i_ and more negative values as [Cl^−^]_i_ falls with increasing time in the methanesulfonate bath, suggests that the contribution of Cl^−^ permeability to the observed currents is a minor one.

The initial seal resistance of 4.8±1.4 GΩ was maintained during deoxygenation, and was recorded at 3.7±0.9 GΩ just before loss of seal. Mean patch duration was 5.3±1.1 min. Inclusion of 1 µM GsMtx-4 in the pipette prevented activation by deoxygenation (Figure 5C). Thus, the deoxygenation-activated cation permeation pathway of SS cells revealed in cell-attached patch configuration conducts Ca^2+^.

### Deoxygenation Activates a Nonspecific Cation Conductance in Cell-Attached Patches of Human SS Red Cells

**Figure 6:**
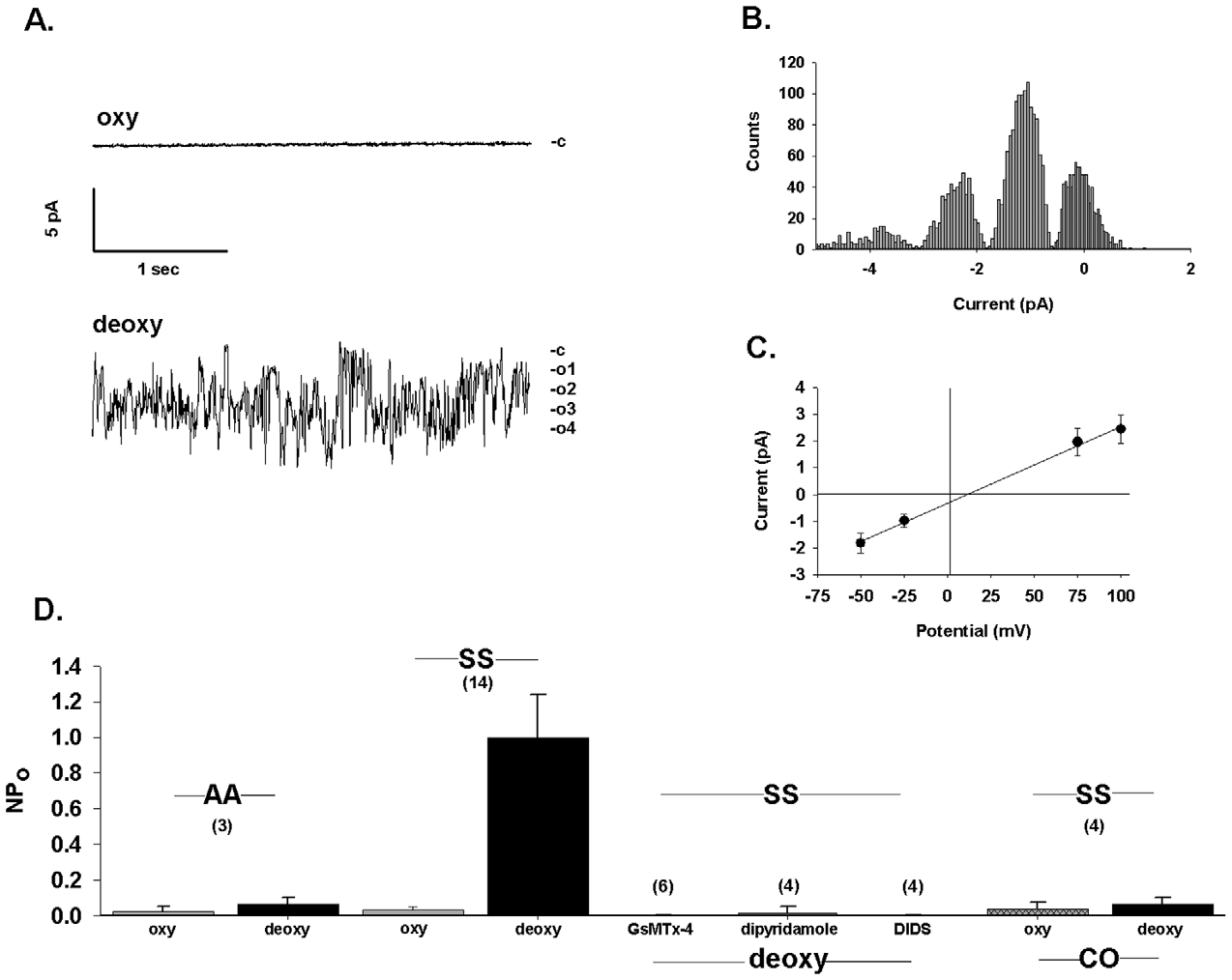
Deoxygenation activates a cation-permeable conductance in human SS cells. A. Current traces recorded from an individual SS red cell patch of 4 GΩ initial seal resistance before (upper trace, oxy) and 2 min after deoxygenation (lower trace, deoxy). Symmetrical pipette and bath solutions contained (in mM) 150 Na methanesulfonate, 10 Na EDTA, and 10 Na HEPES, pH 7.4. Holding potential was −Vp = +50 mV. Open states are at right. Tight seal recording continued under deoxygenated conditions for 8 min beyond the “deoxy” trace shown. Total patch duration was 14 min 18 sec. B. Amplitude histogram from 5 min recording in deoxygenated conditions showing the presence in the panel A patch of at least three equally spaced conductance levels of 1.2±0.3 pA magnitude (mean ± s.d.), consistent with up to four channels in the patch. Estimates of the mean Gaussian fit in the histogram were made with the Simplex least squares method (pCLAMP). C. Current-voltage relationship from the patch shown in panel A, with chord conductance of 29 pS. Mean ± s.e.m. for fit of the amplitude histogram. D. NPo in AA red cells (leftmost two bars) and in SS red cells recorded at −Vp = −50 mV, first in room air and subsequently in deoxygenated conditions (leftmost 4 bars). NPo was measured in on-cell patches of additional SS cells before (not shown) and after deoxygenation in the presence of pipette solution containing GsMTx-4 (1 µM), dipyridamole (100 µM), or DIDS (100 µM), as indicated. Additional cells pretreated with CO prior to on-cell recording were recorded first in oxygenated and then subsequently in deoxygenated conditions (rightmost two bars). The drugs and the pretreatment with CO prevented deoxygenation-induced activation of conductance. Values are means ± s.e.m. for (n) red cells.

Psickle activated by deoxygenation has been characterized by nonspecific cation permeability. We therefore recorded deoxygenation-activated conductance in on-cell patches of SS cells with Ca^2+^-free Na methanesulfonate in both pipette and bath. After switching the room-air equilibrated bath perfusate to perfusate equilibrated with and flushed with 100% N_2_ (arrow), the initially quiescent patch shown in Figure 6A exhibited within 5 seconds a gradually increasing noisy conductance activity that reached steady state within ∼20 sec. In this representative patch, unitary currents of −1.2±0.3 pA (Figure 6B) exhibited a slope conductance of 29 pS (measured beween −Vp = −50 and  = 100 mV) with reversal potential of +11 mV (Figure 6C) and a dwell time of 2 msec (not shown). The mean chord conductance from 14 similar deoxygenation experiments was 27±1 pS (measured between −Vp = 0 and −Vp = −50 mV). Deoxygenation increased NPo at −V_p_ = −50 mV from 0.03±0.02 to 1.00±0.24 (n = 14, P = 0.002). This contrasted with AA cell-attached patches (Figure 6D), in which deoxygenation increased NPo from 0.025±0.024 only to 0.067±0.034 (n = 3, P>0.05). All cells on which stable giga-ohm seals were established and maintained responded to deoxygenation with increased conductance. Mean on-cell patch seal duration in these conditions was 18±11 min. Initial seal resistance of 4.6±0.8 GΩ in room air was maintained under deoxygenated conditions at levels of 3.4±0.7 GΩ prior to seal loss.

The measured inward current could represent cellular Cl^−^ efflux in addition to Na^+^ influx from the pipette, but the near-zero reversal potential (E_rev_) in the absence of pipette chloride strongly supported nonspecific cation-selectivity of this deoxygenation-activated current. The absence of inward current when the pipette contained NMDG chloride further supported the predominant contribution of cation conductance (n = 3, not shown). These data and those of Figure 5 are the first to document deoxygenation-activated conductance in on-cell patch records of individual human SS cells transitioning from room air to hypoxic conditions.

### Pharmacological Properties of Deoxygenation-Induced Conductance Activation in Cell-Attached Patches of SS Red Cells

As was found for deoxygenation-activated Ca^2+^ elevation, inclusion in the pipette of 1 µM GsMTx-4 prevented the deoxygenation-induced increase in cation conductance (NPo 0.003±0.003, n = 6; Figure 6D). GsMTx-4 treatment did not alter red cell shape [[37]; and data not shown].

Previous reports of deoxygenation-induced cation fluxes in SS red cells showed partial inhibition by dipyridamole [32] and DIDS [38]. Inclusion of 100 µM dipyridamole in the patch pipette solution also prevented deoxygenation-induced activation of SS red cell membrane patch cation conductance. Normoxic NPo of 0.00 was unchanged at 0.004±0.002 after deoxygenation (n = 4, P>0.05. not shown). Inclusion of 100 µM DIDS in the pipette similarly blocked conductance activation by deoxygenation, with respective normoxic and hypoxic NPo values of 0.002±0.002 and 0.001±0.001 (n = 4, not shown). Dipyridamole and DIDS have been traditionally treated as nonspecific chloride channel blockers, but they have not been studied as inhibitors of mechanosensitive channels. GsMTx-4 has not been previously reported to block anion channels. 1 µM GsMTx-4 had no effect on KCa3.1 activity in human AA cells treated with 1 µM A23187 in the presence of extracellular Ca^2+^, measured as ^86^Rb influx and as cell shrinkage (n = 3 for each method, not shown). In contrast, the A23187-stimulated ^86^Rb^+^ influx was completely inhibited by the highly specific KCa3.1 inhibitor charybdotoxin (50 nM) [39]. Moreover, A23187-stimulated cell shrinkage was completely inhibited by the moderately specific KCa3.1 inhibitor, clotrimazole (10 µM, n = 3, data not shown; [40]).

The deoxygenation-activated flickery cation conductance observed in human SS cells contrasted with the prolonged channel openings produced by 5 µM LPA (Figure S2A). In SS cells, LPA increased NPo 16-fold, from 0.04±0.03 to 0.82±0.18 (n = 8, p<0.001; Na methanesulfonate in bath and pipette, −Vp = −50 mV; Figure S2B). LPA-induced cation conductance was 96% inhibited by 100 uM dipyridamole in the pipette, and 97% inhibited by 1 µM GsMTx-4 in the pipette (both n = 4).

### Carbon Monoxide (CO) Exposure Prevents Activation of Cation Conductance by Deoxygenation in Cell-Attached Patches on SS Cells

Deoxy-HbSS polymerization is prevented by CO liganding with heme, stabilizing HbS tetrameric structure [41]. As shown in Figure 6D, prior CO exposure of SS cells prevented activation of conductance in on-cell patches upon deoxygenation. The normoxic NPo of 0.0377±0.0375 in CO-pretreated cells did not increase upon deoxygenation (NPo = 0.0625±0.0375, n = 4, P>0.05). Thus, prevention of deoxygenation-induced HbS polymerization was associated with inhibition of deoxygenation-stimulated channel activity.

### Deoxygenation Activates Whole Cell Conductance in Nystatin-Permeabilized Patches of Mouse SAD Sickle Red Cells and Human SS Red Cells

**Figure 7:**
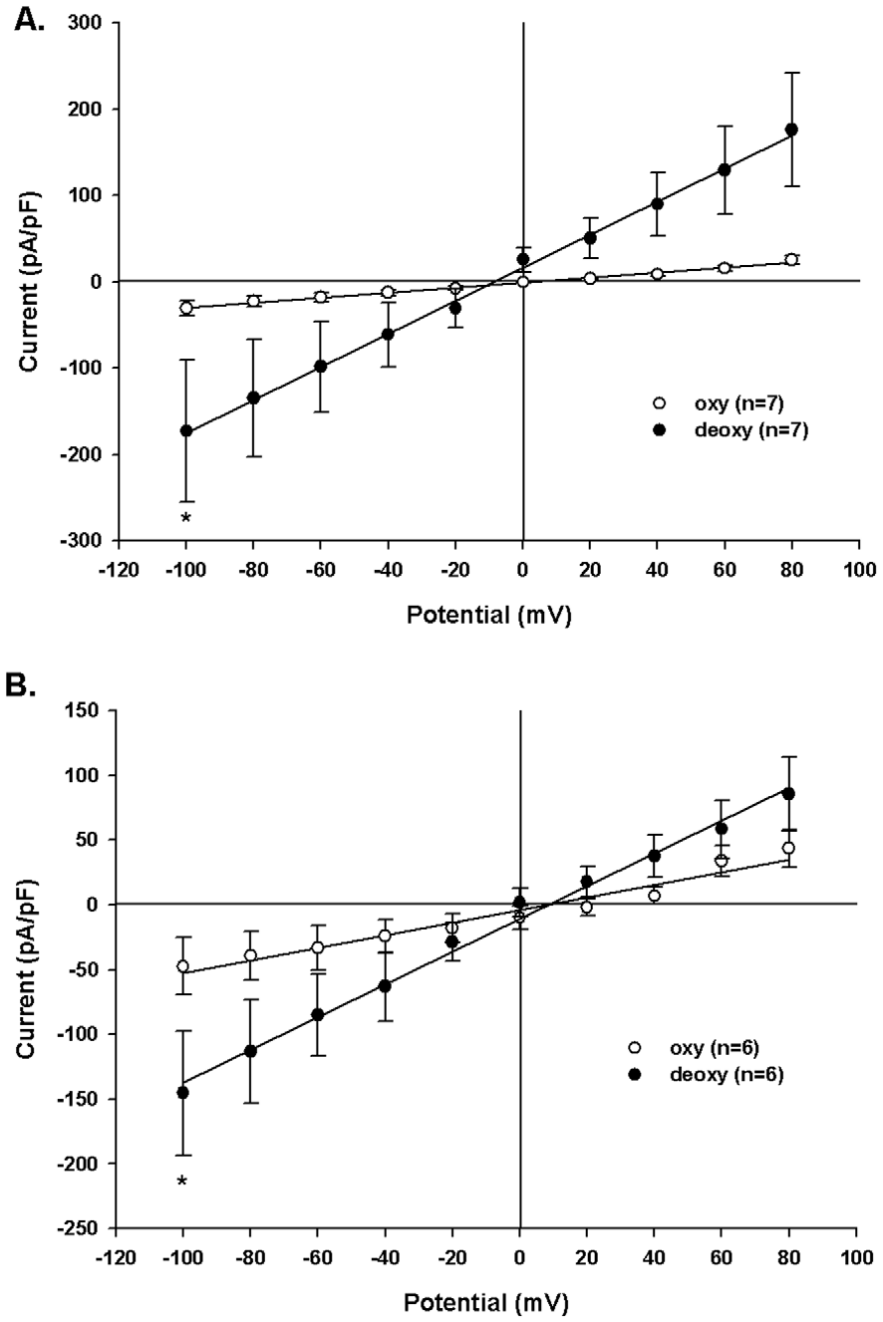
Deoxygenation increases whole cell currents in human and SAD mouse sickle red cells. A. Capacitance-normalized whole cell currents in nystatin-permeabilized patches on intact SAD mouse red cells recorded first in room air (oxy) and then in deoxygenated conditions (deoxy; *, p = 0.016, Wilcoxon; n = 7). B. Capacitance-normalized currents in nystatin-permeabilized patches on intact human SS cells recorded first in room air (oxy) and then in deoxygenated conditions (deoxy; *, p = 0.031, Wilcoxon; n = 6). In both cell types, symmetric pipette and bath solutions contained 150 mM Na methanesulfonate. Values are means ± s.e.m.

Browning et al. showed that deoxygenation-activated currents recorded from human SS cells in conventional whole cell configuration were larger than currents recorded from different SS cells in room air [16]. To extend our on-cell patch results, we examined whole cell cation currents using the nystatin-permeabilized patch configuration in individual cells monitored sequentially in room air followed by deoxygenation, with symmetric Na methanesulfonate in the pipette and the bath. SAD red cells (Figure 7A) increased inward current at −100 mV holding potential from −31±9 nA in room air to −173±82 nA after deoxygenation (n = 7, p = 0.016). Human SS cells (Figure 7B) increased inward current at −100 mV from −48±22 nA in room air to −145±48 nA after deoxygenation (n = 6 p = 0.031). In both cell types, deoxygenation-induced currents were ohmic.

### Kcnn4 Is Not Required for Deoxygenation-Induced [Ca^2+^]_i_ Elevation

**Figure 8:**
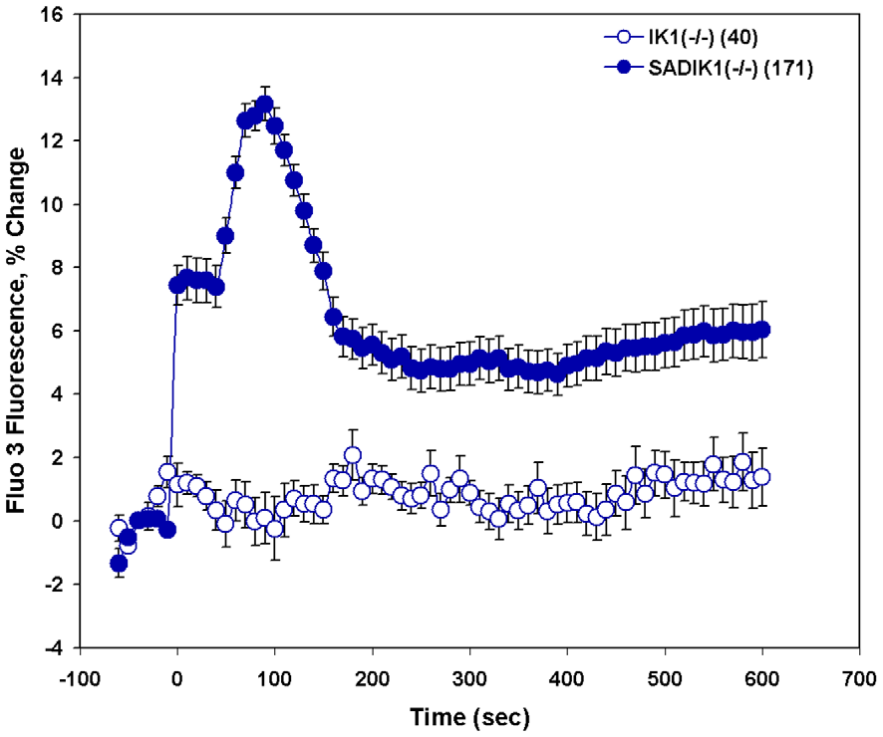
Deoxygenation elevates [Ca^2+^]_i_ in SAD sickle red cells in the absence of Kcnn4/KCa3.1/IK1 “Gardos channel”. Fluo-3-loaded SAD red cells were subjected to deoxygenation at t = 0. Whereas red cells with normal mouse hemoglobin that lacked KCa3.1 [IK1(−/−)] showed no change in [Ca^2+^]_i_, SAD red cells lacking KCa3.1 [SAD/IK1(−/−)] responded to deoxygenation with a substantial increase in [Ca^2+^]_i_ that later fell to a sustained value ∼50% of peak levels. Values are means ± s.e.m. for (n) red cells from two mice studied in two experiments.

Deoxygenation-induced elevation of [Ca^2+^]_i_ might require or be sustained by membrane hyperpolarization caused by KCa3.1 activation. Moreover, the elevated Fluo-3 fluorescence intensity suggestive of elevated [Ca^2+^]_i_ could be influenced by KCa3.1-mediated cell shrinkage of later onset. However, the hypoxia-induced 17% increase in Fluo-3 fluorescence intensity within 2–3 min in human SS red cells (Fig. 4B) is greater than can be simply explained by SS discocyte shrinkage in Cl^−^ medium containing 1 mM Ca^2+^ during 3 min anoxia at 37°C (Figure 6 in [35]). We nonetheless examined the response to deoxygenation of red cells from SAD/*kcnn4^−/−^* mice genetically lacking the Kcnn4/IK1/KCa3.1 K^+^ channel. The normal MCV reduction elicited in wildtype mouse red cells by exposure to A23187 in Ca^2+^-containing medium [39] was abolished in red cells from both *kcnn4^−/−^* mice [6] and from SAD/*kcnn4* ^−/−^ mice (Shmukler and Alper, data not shown). The absence of KCa3.1 in otherwise normal mouse red cells had no effect on the lack of deoxygenation-sensitive [Ca^2+^]_i_ elevation. In contrast, SAD/*kcnn4* ^−/−^ red cells elevated [Ca^2+^]_i_ in response to deoxygenation (Figure 8), as did SAD red cells (Figure 2D) with intact Gardos channel activity [39], [6]. However, shortly after achieving peak [Ca^2+^]_i_, SAD/*kcnn4^−/−^* red cells exhibited a fall in [Ca^2+^]_i_ to a value (Figure 8) approximately 50% that of SAD cells (Figure 2D). This lower plateau value may represent reduced driving force for Ca^2+^ entry through the Psickle-like deoxygenation-induced permeability pathway in the less hyperpolarized state of cells lacking KCa3.1.

## Discussion

The dehydrated state of human and mouse sickle erythrocytes reflects pathological activation of the dominant K^+^ efflux pathways, KCa3.1 (Gardos channel) and the SLC12 K-Cl cotransporters [2], [9], [4]. The deoxgenation-activated entry pathway for the Ca^2+^ that activates KCa3.1, sometimes called Psickle, has been detected as deoxygenation-activated net cation fluxes in human SS cells [42] and in mouse erythrocytes overexpressing human α^H^ and β^S^ globin chains [43]. Psickle has also been detected in human sickle cells as unidirectional radioisotopic cation fluxes [44], [45], [38], [32] and as deoxygenation-activated Gardos channel activity measured by flow cytometry or osmotic fragility assays [35], [34]. Psickle-like activity has also been detected in a comparison of whole cell currents recorded in human SS cells in room air or in deoxygenated conditions [16].

We have reported a deoxygenation-activated, Ca^2+^-permeable, nonspecific cation conductance in both whole-cell and cell-attached patch clamp records from human SS cells and from red cells of two mouse models of sickle disease. The deoxygenation-activated conductance in SAD mouse sickle cells was reversible upon re-oxygenation. We have also demonstrated by fluorimetry deoxygenation-activated elevation of [Ca^2+^]_i_ in single human SS red cells and mouse sickle cells. Deoxygenation-activated conductance and [Ca^2+^]_i_ elevation both were inhibited by GsMTx-4 and by pretreatment with CO, strongly suggesting a requirement for HbSS polymerization in the activation of this permeability pathway. Dipyridamole and DIDS also prevented activation of the conductance. The initial rate of [Ca^2+^]_i_ elevation and its peak were independent of KCa3.1 expression in deoxygenated mouse SAD cells. Several of these properties shared by human and mouse sickle cells are consistent with those of Psickle.

### Comparison of Deoxygenation-Induced Human SS Red Cell Currents Recorded in Cell-Attached and Whole-Cell Configurations

On-cell patches were established in room air, such that currents were recorded first in room air and then from the same patches after deoxygenation. Deoxygenation induced inward Na^+^ and Ca^2+^ currents (Figs. 5 and 6) in all stable cell-attached patches on human SS cells. Deoxygenation-induced currents were completely prevented by 1 µM GsMTx-4, 100 µM DIDS, and 10 µM dipyridamole. Though the data was consistent with activation of nonspecific cation current, a contribution of Cl^−^ current was not ruled out.

Browning et al. compared classical whole-cell patch currents in separate sets of cells under steady-state oxygenation or deoxygenation conditions of symmetrical NaCl [16]. The elevated whole-cell current measured in deoxygenated SS cells was inhibited 20% by DIDS and 42% by Zn^2+^. However, deoxygenation did not increase whole cell inward current measured in Na bath with NMDG in the pipette. Whole-cell current measured in symmetrical [Ca^2+^], was also not increased by deoxygenation [16]. In that study, the whole-cell configuration required patch rupture of previously deoxygenated SS cells, likely imposing transient, high mechanical stress on a rigidified cell membrane. The whole cell configuration employed also diluted cellular HbSS and other cytosolic components, with uncertain effects on membrane-associated HbSS. The mechanical stress experienced by those SS cell membranes exposed not to HbSS-containing cytosol but to pipette solution likely differed from that imposed by deoxygenation on intact sickle red cells with a previously established tight pipette seal.

Whole cell currents recorded in nystatin-permeabilized on-cell patches of human SS and of mouse SAD sickle red cells also demonstrated stimulation upon deoxygenation (Figure 7). The whole cell conductances estimated from nystatin-permeabilized patch records from oxygenated and deoxygenated human SS cells of 135 µm^2^ nominal surface area were 1.0 x 10^−3^ µS cm^−2^ and 2.4×10^−3^ µS cm^−2^, respectively. SAD mouse red cells with nominal surface area of 90 µm^2^ exhibited respective values of 1.3 ×10^−3^ µS cm^−2^ and 9.6×10^−3^ µS cm^−2^ (Figure 7). These conductances are 2 orders of magnitude higher than previously estimated in intact human red cells from valinomycin-limited K^+^ efflux or cell volume change, or from voltage-sensitive fluorescent dyes [46]. This discrepancy between conductances measured in human red cell patch clamp experiments and those measured in ionophore-permeabilized intact human red cells has been previously noted for both anion and cation conductances [47], [16], [46], and remains unexplained.

### Prevalence of Deoxygenation-Activated Currents among SS Cells

The prevalence of Psickle among sickle erythrocytes has been debated. Lew et al. reported a stochastic dehydration response to deoxygenation of sickle cells [35] with only ∼10–45% of cells exhibiting reduction of cell volume as measured by light scattering after up to 45 min of cyclic deoxygenation-reoxygenation. In contrast, among the ∼15% of attempted patches that yielded stable, gigaohm seals in our study, all cells exhibited deoxygenation-activated currents. Browning et al. selected previously deoxygenated sickle cells of irregular morphology for their whole cell patch clamp experiments [16], and selected for analysis only those currents that remained stable in symmetrical Na^+^ conditions for≥6 min. Both the current and previous [16] patch clamp studies may have selected for recording only cell subsets which, among the unselected population studied in the light scattering experiments [35], might have been susceptible to deoxygenation-induced dehydration. Indeed, the stochastic deoxygenation-induced dehydration of SS cells is reflected in the distribution of single cell values of deoxygenation-induced [Ca^2+^]_i_ elevation (Figs. 2D, 3D, 4B), which included cells whose [Ca^2+^]_i_ did not increase in response to the stimulus (Figure S1). The cell age-dependent heterogeneity of Gardos channel activity in AA cells [48] likely also increases the heterogeneity of deoxygenation-induced SS cell dehydration. In contrast, human AA cell dehydration by the erythroid voltage-dependent nonselective cation conductance was uniformly distributed [49].

The complete inhibition of deoxygenation-induced currents in individual patched cells by DIDS and dipyridamole similarly contrasts with the reported partial inhibition of Psickle fluxes by these drugs [15], [9]. The contrast likely reflects a similar sample bias inherent to establishment of stable, tight seals for analysis of patch clamp recordings.

### Ion Selectivity of Deoxygenation-Activated Currents

Psickle is defined by its permeability to Ca^2+^ and monovalent cations. However, its cation-anion selectivity remains undefined. Browning et al. reported [16] that deoxygenation of human SS cells in symmetrical NaCl solutions did not change whole-cell reversal potential (E_rev_). Moreover, asymmetric substitution of pipette Na^+^ with NMDG^+^ shifted E_rev_ minimally in either oxygenated or deoxygenated conditions (without a calculated correction for altered seal resistance). Deoxygenation-induced whole-cell current in human sickle cells was concluded to carry anions as well as cations, and had non-negligible NMDG^+^ permeability [16]. NMDG^+^ permeability also characterized the “Pcat” pathway of senescent human AA red cells [50].

The ionic conditions of our on-cell patch experiments could not rule out a contribution of Cl^−^ conductance to the observed deoxygenation-activated currents, but suggested (Figure 1C) such a contribution is minor. Deoxygenation nonetheless increases human SS cell permeability to many low molecular weight solutes [51], suggesting that deoxygenation either regulates multiple transport pathways or one or more pathways of unusually low selectivity. The noisy character of deoxygenation-activated currents in our on-cell patch records resembles previously published patch records of ligand-induced nonspecific cation conductance in AA red cells [52], [47], and could be intrinsic to Psickle. Alternatively, the noisy signal may include more than one type of hypoxia-activated channel.

### What Is the Relationship between Deoxygenation-Activated Conductances and Previously Reported Erythroid Cation Conductances?

Deoxygenation increases hemoglobin auto-oxidation and generation of reactive oxygen species at the inside of the red cell membrane [53], a process exacerbated in sickle red cells. Psickle and the deoxygenation-activated Ca^2+^-permeable cation conductance reported here may arise from oxidative, proteolytic, or other modifications of proteins present in AA red cells, warranting consideration of modified native AA cell conductances as possible contributors to Psickle.

Normal human red cells respond to oxidative stress with increased Cl^−^-dependent cation channel activity [54], which may be further increased in sickle cells [53]. A depolarization-activated 30 pS cation channel of AA cells [55]; [47] displays voltage-dependence unlike the deoxygenation-activated currents reported in the current work. Lysophosphatidic acid (LPA) and prostaglandin E2 (PGE2) elevate [Ca^2+^]_i_ in AA cells. LPA-induced [Ca^2+^]_i_ elevation is PKC-mediated and sensitive to inhibition by ω-2-agatoxin [56]. These properties, along with the long open and closed times of LPA-induced channel activity and its attendant [Ca^2+^]_i_ increase (10-fold higher than elicited by deoxygenation in human SS cells; Figure S2), all differ from the deoxygenation-activated responses reported in the current work. The Ba^2+^-insensitive, PGE2-induced Ca^2+^ entry pathway remains unidentified [52], but erythrocytes themselves release PGE2 in response to the erythroid cation channel activators, hypertonicity and chloride depletion. The red cell [Ca^2+^]_i_ elevation induced by LPA, PGE2, and other agents stimulates erythroid suicidal cell death (eryptosis) by increasing annexin V externalization, promoting erythrophagocytosis by the reticuloendothelial system without lytic release of toxic free hemoglobin [57].

Missense mutations of Band 3/AE1/SLC4A1 can cause cation-leak hereditary stomatocytosis [58], and expression of stomatocytosis-associated AE1 mutants in oocytes has been reported to increase conductive cation transport [59]. Thus, oxidatively modified AE1 has also been proposed as Psickle [16], but further evidence supporting this hypothesis is awaited. A role of mechanosensitive ATP release and paracrine activation of erythroid ionotropic [60] or metabotropic purinergic receptors [61] remains to be investigated in the activation of Psickle and/or associated conductances by deoxygenation.

### Conclusion

The conductance induced by deoxygenation in on-cell patches of human HbSS red cells and of mouse sickle cells is permeable to Na^+^, to Ca^2+^, and to other cytoplasmic cations. The conductance is inhibited by the blocker of mechanosensitive cation channels, GsMTx-4, by DIDS, and by dipyridamole. The inhibitor of deoxy-HbSS polymerization, CO, also prevents activation of the cation conductance by deoxygenation. Moreover, deoxygenation increases whole cell conductance in the nystatin-permeabilized on-cell patch configuration. These properties resemble many of those previously described for the hypoxia-activated permeability of human SS red cells known as Psickle. The deoxygenation-activated conductance is likely responsible for [Ca^2+^]_i_ elevation in human and mouse sickle cells, leading to activation of KCa3.1 (Gardos channel), with consequent acceleration of pathological sickle cell dehydration. However, KCa3.1 is not itself required for hypoxic activation of the Ca^2+^-permeable cation conductance. Future studies should lead to molecular identification of component polypeptide(s) of the deoxygenation-activated cation conductance, and more completely define the mechanism linking its activation to HbS polymerization. Clinically tolerated inhibitors of the deoxygenation-activated cation conductance should synergize with senicapoc in prevention of pathological sickle cell dehydration.

## Materials and Methods

### Ethics Statement

Human SS and AA blood was obtained as discarded, anonymized, laboratory samples under IRB-approved protocols of Children's Hospital and Beth Israel Deaconess Medical Center. Mouse blood was withdrawn by cardiac puncture or from the tail vein into heparinized syringes according to IACUC-approved protocols of Beth Israel Deaconess Medical Center.

### Materials

*Grammastola spatulata* mechanotoxin IV (GsMTx-IV) was purified [24] from *G. spatulata* crude venom (Spider Pharm, Yarnell, AZ) or purchased from Peptides International (Louisville, KY). 4,4′-diisothiocyanatostilbene-2,2′-disulfonic acid (DIDS) was from Calbiochem (San Diego, CA). Other drugs and analytical grade salts were from Sigma or Fluka (St. Louis, MO).

### Blood Cell Preparation

Human SS and AA blood was obtained as discarded, anonymized, laboratory samples under IRB-approved protocols of Children's Hospital and Beth Israel Deaconess Medical Center. After buffy coat removal and 5 washes in standard human red cell wash solution containing (in mM) 150 choline Cl, 1 MgCl, and 10 Tris-MOPS, pH 7.4, red cells were resuspended in storage solution containing (in mM) 145 KCl, 15 NaCl, and 10 HEPES, pH 7.4, then kept at 4°C until used. Human red cells allowed to settle on coverslips were mounted on an inverted microsope in a 200 µl open chamber (WPI, Sarasota, FL) and superfused 15 min at room temperature by bath solution containing (in mM) 150 Na methanesulfonate, 10 Na EDTA, and 10 Na HEPES, pH 7.4.

Blood was withdrawn by cardiac puncture or from the tail vein of C57Bl6/J wildtype mice, SAD sickle mice [5], [17], or Berkeley sickle mice [31] into heparinized syringes according to IACUC-approved protocols of Beth Israel Deaconess Medical Center. Buffy coat-depleted cells were washed 5 times in standard mouse red cell wash solution containing (in mM) 172 choline Cl, 10 sucrose, 10 Na Tris-MOPS, pH 7.4, resuspended in storage solution, and kept at 4°C until use.

Mouse red cells allowed to settle on coverslips were superfused 15 min with bath solution containing (in mM) either 150 mM Na methanesulfonate, 10 Na EDTA, and 10 Na HEPES, pH 7.4; or 140 NaCl, 4 KCl, 1CaCl_2_, 1 MgCl_2_, 10 Na HEPES, pH 7.4. Deoxygenation was achieved by switching superfusate to the same solution gassed ≥30 min prior to the experiment with 100% N_2_, and by flushing of the perfusion chamber with N_2_ during the deoxygenation period. The resulting bath pO_2_ was18 mm Hg as measured by oxygen electrode (WPI). In the experiments of Figure 1, deoxygenated cells were subsequently re-oxygenated by exposure to the same superfusate equilibrated with room air.

Washed human or mouse red cells in solution containing (in mM) 140 NaCl, 5 KCl, 1 CaCl_2_, 1 MgCl_2_, 10 Na HEPES, pH 7.4 were exposed for 1 hr on a rotary platform in a 37°C incubator to 25 ppm carbon monoxide (CO) in 5% CO_2_. CO-exposed cells were then subjected to patch clamp or to calcium imaging experiments within 60 min after return to room air, using the solutions indicated.

### On-Cell Patch Clamp

Borosilicate pipettes (Corning 7052) pulled with a Narishige two stage puller or a Sutter P97 puller and fire-polished to resistances of 10–20 MΩ were front-filled and then backfilled. Symmetric bath and pipette solutions for studies of seal resistance stability during reversibility tests of the deoxygenation-activated conductance in SAD mouse red cells were (in mM) 140 NaCl, 4 KCl, 1 CaCl_2_, 1 MgCl_2_, 10 Na HEPES, pH 7.4. These conditions yielded tight seals in 13% of patch attempts on SAD cells. For study of deoxygenation-induced monovalent cation permeation in the absence of bath Ca^2+^ and Mg^2+^ recorded in SAD and Berkeley mouse cells and in human cells, symmetric bath and pipette solutions contained (in mM) 150 Na methanesulfonate, 10 Na EDTA, and 10 Na HEPES, pH 7.4. These conditions yielded tight seals in 16% of patch attempts on SAD mouse red cells, 26% of attempts on Berkeley mouse cells, and 30% of attempts on human SS cells. For study of Ca^2+^ permeation in human SS cells, bath and pipette solutions contained (in mM) 100 CaCl_2_, 10 Na HEPES, pH 7.4. These conditions yielded tights seals on 44% of attempts. For all conditions tested in human red cells, 70% of tight seals were sustained through the solution change accompanying deoxygenation to allow recording of current activity.

On-cell patch currents were recorded with the Axopatch 1-D amplifier (Axon Instruments/Molecular Devices, Sunnyvale, CA). Holding potential was −Vp  = −50 mV (expressed as the negative of the pipette potential (e.g., equivalent to the intracellular potential with respect to the pipette). To determine current-voltage relationships (I-V curves) in Fetchex or Clampex (PCLAMP, Axon Instruments), the realtime control window in gap-free mode was used to record current traces of 10−30 sec duration at holding potentials ranging from −100 to +100 mV, in 25 mV increments. The bath reference electrode was a silver chlorided wire with a 3 M KCl agar bridge. Data was filtered at 500 Hz, digitized at 2 kHz by Clampex, and analyzed offline by Fetchan and Pstat or by Clampfit subroutines of PCLAMP. Holding potentials in on-cell patch experiments were expressed as −Vp, the negative of the pipette potential.

Nystatin-permeabilized on-cell patch conditions were modified from Mahaut-Smith as previously described [62], [46], using symmetric bath and pipette solutions of 150 mM Na methanesulfonate. These nominal whole cell currents were recorded with the Axopatch 1-D amplifier, and normalized to capacitance as measured by analysis of the current transient elicited by stepping to +100 mV [46]. I-V curves were measured during sequential 20 mV voltage steps of 200 msec clamp duration, between −100 mV and +80 mV. Output was digitized, filtered, and analyzed offline as above.

### Fluorescence Measurements of Cytosolic [Ca^2+^] ([Ca^2+^]_i_)

After removal of plasma and buffy coat by aspiration, human or mouse red cells were washed three times at room temperature and suspended in modified Hank's solution containing (in mM), 137 NaCl, 5.4 KCl, 1 CaCl_2_, 1 MgCl_2_, 0.8 NaK phosphate, 5.6 glucose, and 10 Na HEPES, pH 7.4. Dilute cell suspensions were settled on polylysine-coated coverslips forming the bottom of an open perfusion chamber (2.5 cm diameter, 1 cm depth) and washed again 3 times.

Measurement of relative changes in [Ca^2+^]_i_ by Fluo-3 fluorescence emission was previously validated in intact human [36], [56] and mouse red cells [63]. Attached red cells were loaded in the dark with the fluorescent non-ratiometric Ca^2+^ indicator Fluo-3-AM (10 µM, Molecular Probes, Eugene, OR) at 37°C for 1 hr, then washed and incubated 15 min further to allow de-esterification of intracellular dye. The open perfusion chamber containing dye-loaded cells was mounted on an Olympus IMT-2 inverted epifluorescence microscope equipped with CoolSNAP CCD camera (Photometrics, Tucson, AZ). Fluo-3 in cells imaged through a 60x objective was excited by mercury-xenon illumination passed through a 495ds20 filter. Fluorescence emission (proportional to [Ca^2+^]_i_) from a 535ds20 bandpass filter was collected from all attached single cells within the arbitrarily selected visual field. Emission image acquisition was controlled by a Metafluor digital imaging system (Universal Imaging, West Chester, PA).

Fluo-3 fluorescence from attached cells on each coverslip was recorded 60 sec in room air and then for 10 min after onset of perfusion chamber flushing with humidified 100% N_2_. The normalized increase in Fluo-3 fluorescence in SS cells subjected to this deoxygenation procedure was indistinguishable from that observed during superfusion of cells in a closed perfusion chamber via syringe pump with bath previously bubbled for 60 min with 100% N_2_ (n = 62 cells from 2 SS patients, not shown). The y-axis value of 0% change in Fluo-3 fluorescence intensity represented mean intensity recorded from all cells within the coverslip visual field during the 60 sec prior to deoxygenation.

Intracellular hydrolysis of acetoxymethyl esters generates formaldehyde, which lowers cellular ATP concentration through inhbibition of glycolysis [64], [65], [66]. Total red cell exposure time to acetoxymethyl ester from the start of Fluo-3-AM loading to the end of our fluorescence measurements was 90 min. ATP concentrations in human red cells incubated for 4 hrs under similar conditions, in the presence of glucose without pyruvate supplementation, were shown previously not to fall below 80% of initial values [36].

### Mouse Breeding

C57BL6/J inbred mice and Berkeley sickle mice (*Hba^tm1Paz^ Hbb^tm1Tow^ Tg(HBA-HBBs)41Paz/J*)[31] purchased from JAX (Bar Harbor, ME) were bred and genotyped as recommended by JAX protocol (http://jaxmice.jax.org/strain/003342.html). SAD-1 sickle mice (*Hbb^S/S^ Tg(HBA-HBB-SAD-1*) transgenic for one copy of the hypersickling human β-globin gene carrying the three mutations βS ^6^Val, β S−Antilles ^23^Ile, and β D Punjab ^121^Gln and (in the founder expressed in their red cells ∼19% Hb SAD (a_2_^human^β_2_^SAD^), mouse Hb, and mouse-human hybrid Hb [5], [17], [67] (SAD mice), were crossed with *Kcnn4^−/−^* mice [6] ultimately to generate *Kcnn4^−/−^* progeny carrying the SAD transgene. SAD x *Kcnn4^−/−^* progeny were born at Mendelian ratios, and exhibited no gross phenotypic difference from SAD mice. KCa3.1 genotyping [6] and Hb β-SAD genotyping protocols [5] were as described.

### Statistical Analysis

Statistical analyses were performed with SigmaStat v.2.03 (SSPS, Chicago, IL). Paired or unpaired t-tests, the Mann-Whitney test, or the Wilcoxon rank-order test were used as indicated.

## Supporting Information

Figure S1 A. Changes in Fluo-3 fluorescence intensity in (n) individual human SS red cells 210 sec after deoxygenation (*, P<10–9), and in (n) individual Berkeley sickle mouse red cells 300 sec after deoxygenation (*, P<10–7). 1 µM GsMTx-4 was absent or present as indicated. B. Change in Fluo-3 fluorescence intensity in (n) individual SAD sickle mouse red cells 100 sec after deoxygenation, in the absence and presence of 1 µM GsMTx-4 (P<10–9). The selected time points are those of maximally elevated mean [Ca2+]i as observed in Fig. 2 for SAD mouse cells, in Fig. 3 for Berkeley mouse cells, and in Fig. 4 for human SS cells.
(0.65 MB PDF)

Figure S2 Lysophosphatidic acid activates a cation-permeable channel in human SS red cells. A. On-cell patch recording from a human SS red cell before (upper trace) and after exposure to 5 µM lysophosphatidic acid (LPA, lower trace). Symmetrical bath and pipette solutions contained (in mM) 150 Na+ methanesulfonate, 10 Na EDTA, and 10 Na HEPES, pH 7.4. −Vp = −50 mV. B. LPA-induced increase in NPo was prevented by inclusion of either 1 µM GsMTx-4 or 100 µM dipyridamole in the pipette. Values are means + s.e.m. for 4–8 SS red cells; *, P<0.001. C. LPA elevated [Ca2+]i in human SS red cells, as indicated by Fluo-3 fluorescence increase. Values are means + s.e.m. for 75 SS red cells from 3 subjects examined in two experiments.
(0.77 MB PDF)
