# Supplementary figures and images for: Hypoxia Activates a Ca2+-Permeable Cation Conductance Sensitive to Carbon Monoxide and to GsMTx-4 in Human and Mouse Sickle Erythrocytes
Source: PLoS One. 2010 Jan 15;5(1):e8732. doi: 10.1371/journal.pone.0008732 (PMC2806905; doi:10.1371/journal.pone.0008732)

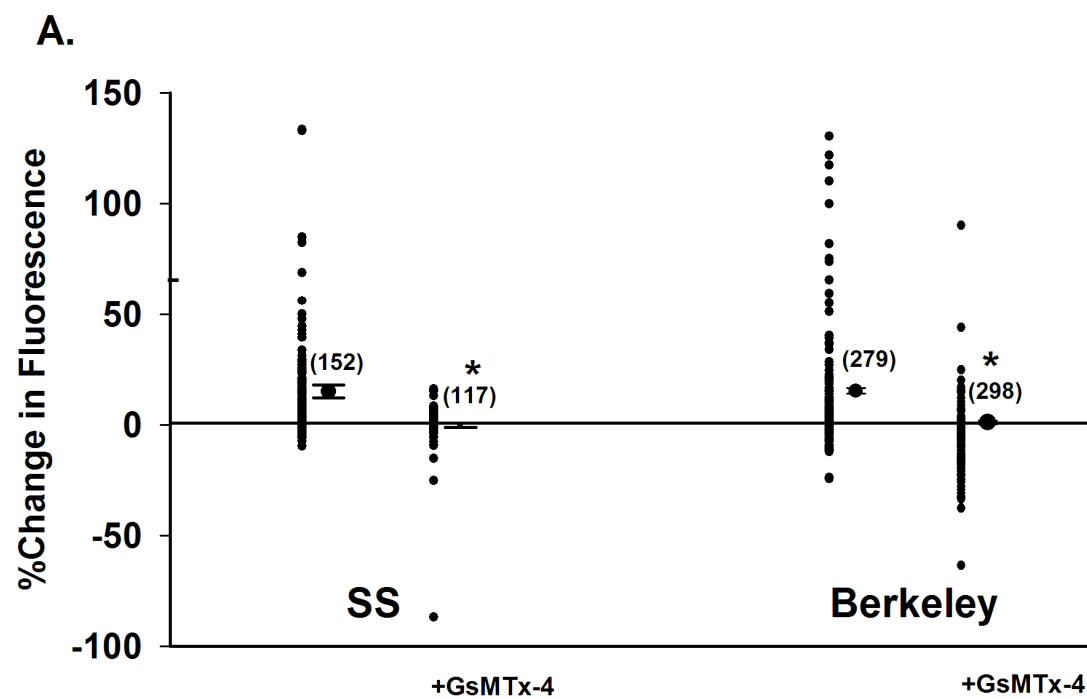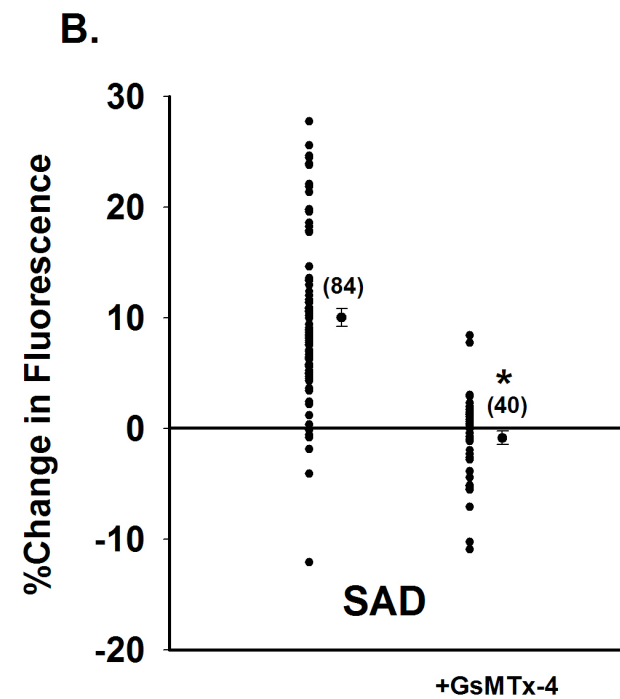

Supplement: Figure S1 — A. Changes in Fluo-3 fluorescence intensity in (n) individual human SS red cells 210 sec after deoxygenation (*, P<10–9), and in (n) individual Berkeley sickle mouse red cells 300 sec after deoxygenation (*, P<10–7). 1 µM GsMTx-4 was absent or present as indicated. B. Change in Fluo-3 fluorescence intensity in (n) individual SAD sickle mouse red cells 100 sec after deoxygenation, in the absence and presence of 1 µM GsMTx-4 (P<10–9). The selected time points are those of maximally elevated mean [Ca2+]i as observed in Fig. 2 for SAD mouse cells, in Fig. 3 for Berkeley mouse cells, and in Fig. 4 for human SS cells. (0.65 MB PDF) [file pone.0008732.s001.pdf]

**A.**

control

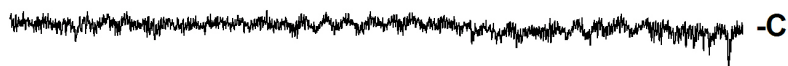

post LPA

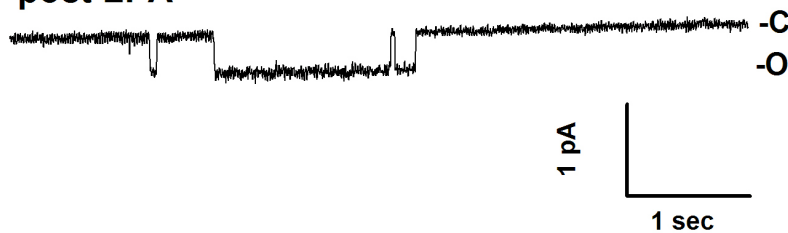**B.**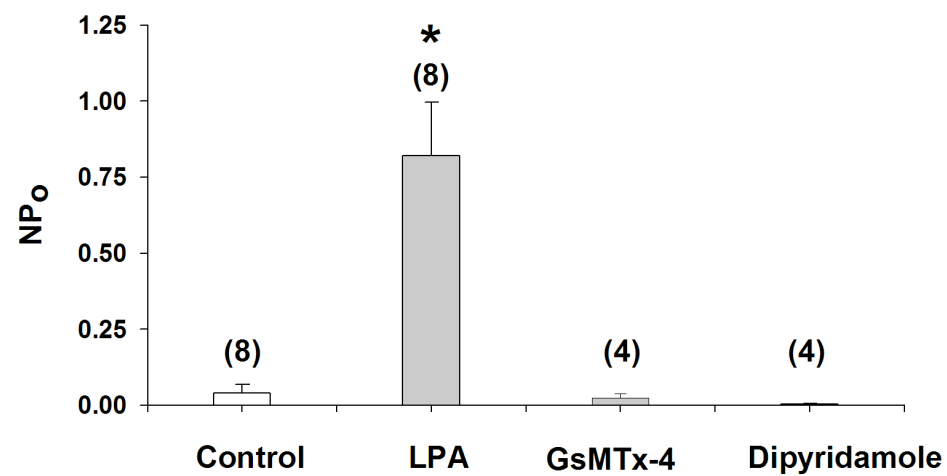**C.**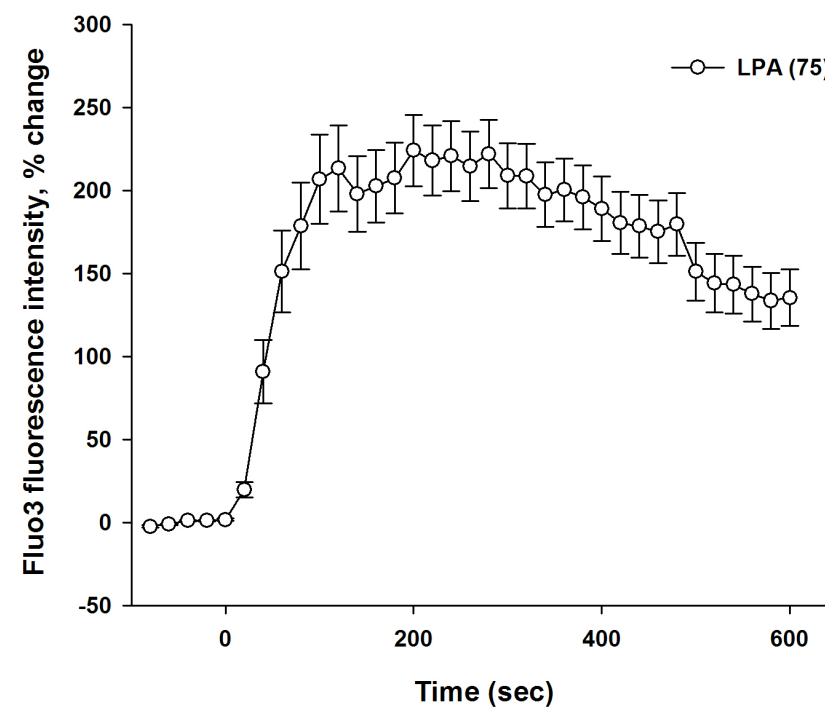

Supplement: Figure S2 — Lysophosphatidic acid activates a cation-permeable channel in human SS red cells. A. On-cell patch recording from a human SS red cell before (upper trace) and after exposure to 5 µM lysophosphatidic acid (LPA, lower trace). Symmetrical bath and pipette solutions contained (in mM) 150 Na+ methanesulfonate, 10 Na EDTA, and 10 Na HEPES, pH 7.4. −Vp = −50 mV. B. LPA-induced increase in NPo was prevented by inclusion of either 1 µM GsMTx-4 or 100 µM dipyridamole in the pipette. Values are means + s.e.m. for 4–8 SS red cells; *, P<0.001. C. LPA elevated [Ca2+]i in human SS red cells, as indicated by Fluo-3 fluorescence increase. Values are means + s.e.m. for 75 SS red cells from 3 subjects examined in two experiments. (0.77 MB PDF) [file pone.0008732.s002.pdf]
